# Elranatamab treatment in a multiple myeloma patient undergoing renal dialysis

**DOI:** 10.46989/001c.116800

**Published:** 2024-05-06

**Authors:** Zoé Van de Wyngaert, Irene Romera-Martinez, Céline Chedeville, Paolo Musiu, Souhila Ikhlef, Bénédicte Jonca, Mohamad Mohty, Florent Malard

**Affiliations:** 1 Sorbonne Université ; Centre de Recherche Saint-Antoine INSERM UMRs938; 2 Service d’Hématologie Clinique et de Thérapie Cellulaire, Hôpital Saint Antoine, AP-HP, Paris, France; 3 Service de médecine nucléaire Tenon Hospital

**Keywords:** dialysis, elranatamab, BCMA, multiple myeloma, bispecifics

## Abstract

We present the case of a dialyzed patient with relapsed IgA and lambda free light chain multiple myeloma treated with elranatamab. Despite end-stage renal impairment, the treatment with anti-B cell maturation antigen (BCMA)xCD3 bispecific antibody proved to be feasible, without unexpected side effects. Increased attention to infectious risk is crucial for these doubly fragile patients.

## Case report

A 79-year-old female patient was diagnosed with IgA and lambda free light chain (FLC) multiple myeloma (MM) in June 2018. At diagnosis, she had anemia, hypercalcemia, renal impairment and diffuse bone marrow involvement, with stage 3 R-ISS and translocation t(4;14). She received nine cycles of bortezomib, melphalan and prednisone as a first-line therapy between July 2018 and August 2019, and achieved a complete response.

She experienced first relapse in February 2020, with an IgA M-spike of 26 g/L, lambda FLC at 1,450 mg/L, and a large plasmacytoma on the fifth left rib extending into soft tissues. A second line with daratumumab, lenalidomide and dexamethasone was started in March 2020. The patient was refractory, and a biological progression was seen after two cycles. A third line with daratumumab, pomalidomide and dexamethasone was initiated in May 2020, but it proved ineffective as the patient had, again, a biological progression after two cycles. A fourth line of treatment with carfilzomib, pomalidomide and dexamethasone (KPD) was started in July 2020. She finally achieved a complete response with KPD, which was sustained for two years.

Simultaneously, she had a slow deterioration of renal function, and became dependent on dialysis from September 2020.

**Figure 1.**
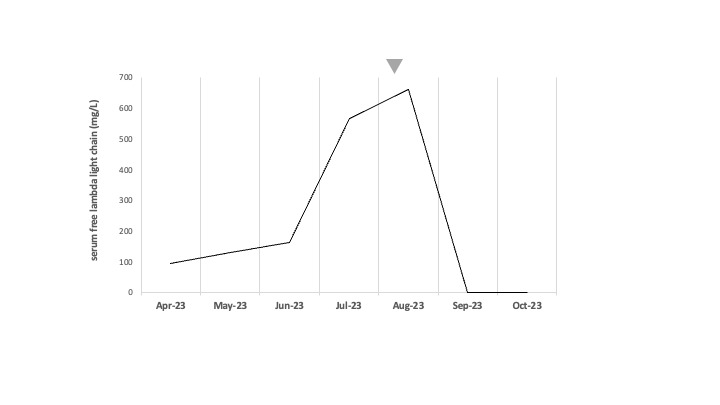
Evolution of serum free lambda light chain levels (mg/L) over time. The triangle corresponds to the initiation of elranatamab

**Figure 2.**
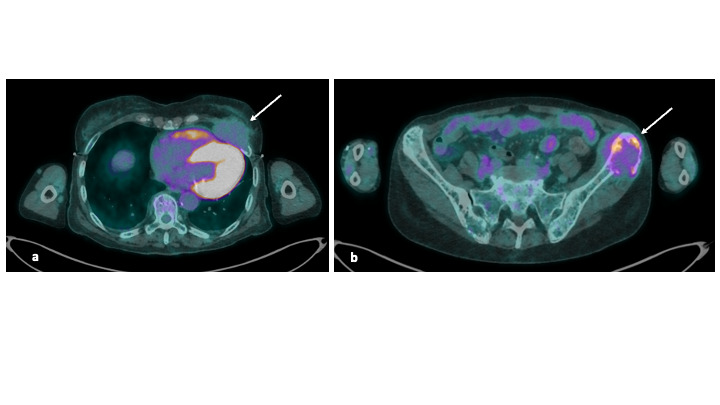
^18-^FDG-PETscan, showing left rib (a) and left iliac crest (b) plasmacytomas, invading soft tissues (white arrows)

From August 2022, she had a biological relapse with a gradual increase in lambda FLC (Figure 1). In July 2023, lambda FLC was at 566 mg/L, the patient had moderate anemia at 11.8 g/dL with erythropoietin treatment, no hypercalcemia, and no bone pain. The gammaglobulin level was 3.7 g/L. The ^18-^FDG PET-scan showed hypermetabolic bone lesions located in the sternum and ribs, and plasmacytomas on the 5th left rib and the left iliac crest. A pelvic CT-scan showed that the iliac lesion had invaded soft tissues and measured five centimeters (Figure 2).

Treatment with the anti-BCMAxCD3 bispecific antibody elranatamab was started on August 2023. Prophylactic antibiotics with cotrimoxazole and valacyclovir were continued. The treatment began with a standard ramp-up phase, after premedication with dexamethasone, polaramine, and paracetamol. Injections were systematically performed after a dialysis session (first dose was given at 12 mg on day 1, second dose at 32 mg on day 6). The patient experienced a grade I cytokine release syndrome (CRS) after the second dose, which persisted for more than 72 hours, prompting the administration of tocilizumab infusion (8 mg/kg) on day 9. A third dose was given at 76 mg on day 13, after CRS resolution. She experienced no immune effector cell-associated neurotoxicity syndrome (ICANS) and no significant cytopenias.

**Figure 3.**
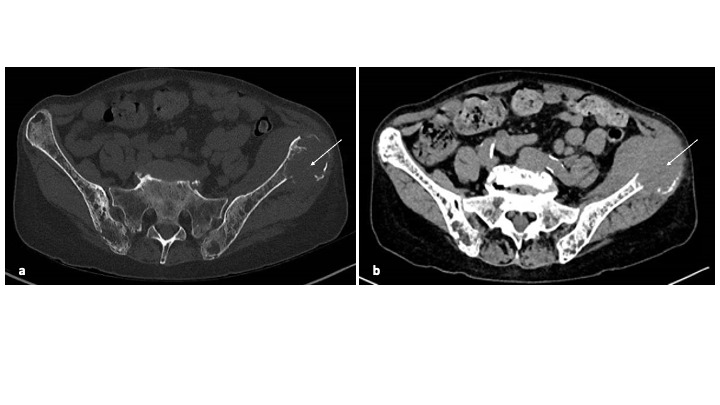
Pelvic CT-scan after two weeks of treatment, in bone window (a) and soft tissue window (b), showing a large lytic lesion of the left iliac crest invading soft tissues (white arrow)

After two weeks of treatment, the patient was readmitted due to sudden left hip pain. Lambda FLC showed a slight increase to 660 mg/L. The pelvic CT-scan revealed a discreet progression of the left iliac crest lesion, invading soft tissues and measuring 6 cm (Figure 3). It was decided to continue the treatment. The patient also underwent left hip irradiation (8-Gray in one fraction) for pain relief on day 29 after the first dose. After one month of elranatamab treatment, a complete response was finally achieved, with FLC normalization (Figure 1).

**Figure 4.**
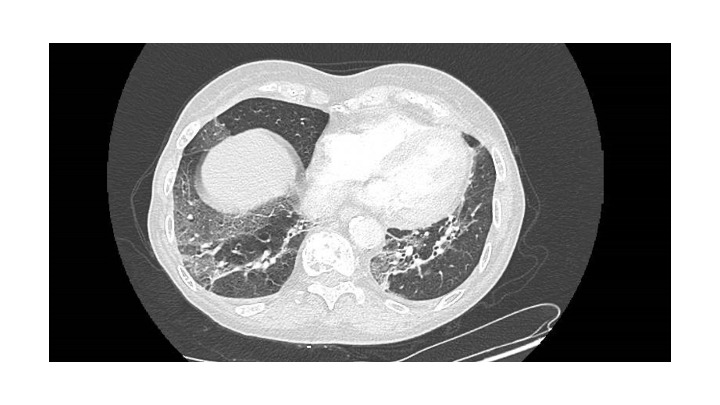
Thoracic CT-scan, showing SARS-CoV-2 pneumonia with severe involvement, affecting between 25 and 50% of the lung parenchyma, with associated atelectasis bands

Subsequently, after two months of treatment, the patient developed oxygen-requiring SARS-CoV-2 pneumonia in October 2023, with extensive radiographic involvement (Figure 4), from which she recovered slowly. She remains in complete response at the latest follow-up, four months after treatment initiation.

## Discussion

This is the first report of a dialyzed patient receiving an anti-BCMAxCD3 bispecific antibody. In the phase 1-2 trials of teclistamab and elranatamab, the minimal estimated glomerular filtration rate (eGFR) required was 40 and 30 mL/min/1.73m^2^, respectively.^1,2^

Although data are scarce due to patients with severe renal impairment usually being excluded from clinical trials, there is no evidence of significant renal clearance of immunotherapies that are either antibody or cell-based. Furthermore, there is no significant incidence of renal toxicity attributable to these treatments.

One small series reported on 13 patients with severe renal impairment (eGFR ≤15mL/min/1.73m^2^), including six dialysis-dependent patients, treated with daratumumab without dose adjustments.^3^ There was no significant or unexpected toxicity. One patient had congestive heart failure related to excessive fluid infusion combined with the use of high doses of dexamethasone. Subcutaneous administration of such therapeutics should limit this problem. Interestingly, dialysis independence was achieved in three patients.

Data issued from the DREAMM-2 study of belantamab-mafodotin, showed that patients with mild or moderate renal impairment (eGFR 30–90 mL/min/1.73 m^2^) had similar response rates to those with normal renal function, although the authors observed slightly higher rates of anemia and thrombocytopenia in the former.^4^ In a real-life cohort of 36 patients treated with belantamab-mafodotin, two patients had creatinine >2.5 mg/dL and one patient was on dialysis ; all three received full dose belantamab-mafodotin, with no significant toxicity.^5^

A recent review reported outcomes of 28 MM patients with renal impairment (eGFR≤50mL/min/1.73 m^2^) treated with the anti-BCMA CAR-T cell therapy idecabtagene vicleucel (ide-cel), including 11 with severe renal impairment (eGFR<30 mL/min/1.73 m^2^, including one on dialysis). Patients had a higher incidence of short-term cytopenia, but, otherwise, the safety (incidence of CRS, ICANS) and efficacy profiles were similar to those with normal renal function.^6^

Although no data are yet available for bispecific antibodies in patients with renal impairment, given the similar pharmacokinetic properties of these IgG-based antibodies, we do not expect major differences in safety and efficacy than in patients with normal renal function. Furthermore, from another field of medicine, in a current phase 1 trial, anti-BCMAxCD3 bispecific antibodies are being given to highly sensitized hemodialysis patients to reduce human leukocyte antigen (HLA) antibody levels before kidney transplant (NCT05092347^7^), suggesting the feasibility of such treatment in hemodialysis patients.

In our patient, we decided to systematically administer the treatment after a dialysis session, to limit the risk of elimination of the product. We did not observe severe or atypical side effects after treatment. Grade 1 CRS is quite frequent, as it is observed in 55 to 72% of patients treated with anti-BCMAxCD3 bispecific antibodies.^1,2^

The infectious risk is significant in patients treated with anti-BCMAxCD3 bispecific antibodies.^8–10^ Early supplementation with polyvalent immunoglobulins (Ig) during the first months of treatment has been recommended.^8^ Unfortunately, our patient developed oxygen-requiring SARS-CoV2 pneumonia, despite up-to-date vaccination and positive serology. This emphasizes the need for preventive measures, especially among comorbid patients. Polyvalent Ig supplementation was implemented after this episode. It was also decided to resume elranatamab treatment with bi-weekly injections. Indeed, it has been suggested that spacing injections might reduce the incidence of infections.^1,11^

Efficacy was observed after the first cycle, with the patient achieving complete response. It is noteworthy that after two weeks of treatment, the patient presented with a pseudo-progression with an increase in bone pain and a discreet enhancement in the size of a plasmacytoma on CT-scan. This might be related to the local inflammatory reaction induced by bispecific antibodies.^12^ This fact underscores the importance of not hastily concluding a treatment failure.

In conclusion, elranatamab treatment proved effective in this quadri-refractory patient with t(4;14), and it was feasible despite the situation of end-stage renal impairment with dialysis. No unexpected side effects were observed. Special attention must be given to the infection risk, considering dialysis as an additional risk factor.

### Authorship

ZV wrote the first draft of the manuscript, and all authors commented on subsequent versions. All authors contributed to data collection, and patient follow-up. All authors read and approved the final manuscript.

### Statements and Declarations

ZV declares honoraria from BMS, Sanofi, Janssen-Cilag. MM has received research support and lecture honoraria from Adaptive, Amgen, Astellas, BMS, GlaxoSmithKline, Janssen, Jazz, Novartis, Pfizer, Takeda, Sanofi, and Stemline, all outside the scope of this work. FM reports honoraria from Therakos/Mallinckrodt, Janssen, Sanofi, JAZZ Pharmaceuticals, Gilead, Novartis, and Astellas, all outside the scope of this work. The remaining authors declare no competing financial interests.
